# Associations of Chronic Kidney Disease With Dementia Before and After TIA and Stroke: Population-Based Cohort Study

**DOI:** 10.1212/WNL.0000000000013205

**Published:** 2022-02-15

**Authors:** Dearbhla M. Kelly, Sarah T. Pendlebury, Peter M. Rothwell

**Affiliations:** From the Wolfson Center for Prevention of Stroke and Dementia, Nuffield Department of Clinical Neurosciences, John Radcliffe Hospital, University of Oxford, UK.

## Abstract

**Background and Objectives:**

Individuals with chronic kidney disease (CKD) appear to be at increased risk of cognitive impairment, with both vascular and neurodegenerative mechanisms postulated. To explore the vascular hypothesis, we studied the association between CKD and dementia before and after TIA and stroke.

**Methods:**

In a prospective, population-based cohort study of TIA and stroke (Oxford Vascular Study; 2002–2012), pre-event and new postevent dementia were ascertained through direct patient assessment and follow-up for 5 years, supplemented by review of hospital/primary care records. Associations between pre-event dementia and CKD (defined as an estimated glomerular filtration rate [eGFR] <60 mL/min/1.73 m^2^) were examined using logistic regression and between postevent dementia and CKD using Cox and competing risk regression models, adjusted for age, sex, education, stroke severity, prior stroke, white matter disease, diabetes mellitus, and dysphasia.

**Results:**

Among 2,305 patients with TIA/stroke (median [interquartile range] age, 77 [67–84] years, 1,133 [49%] male, 688 [30%] TIA), 1,174 (50.9%) had CKD. CKD was associated with both pre-event (odds ratio [OR] 2.04 [95% confidence interval (CI) 1.52–2.72]; *p* < 0.001) and postevent dementia (hazard ratio [HR] 2.01 [95% CI 1.65–2.44]; *p* < 0.001), but these associations attenuated after adjustment for covariates (OR 0.92 [0.65–1.31]; *p* = 0.65 and HR 1.09 [0.85–1.39]; *p* = 0.50). The results were similar when a competing risk model was used (subdistribution HR [SHR] 1.74 [1.43–2.12]; *p* < 0.001, attenuating to 1.01 [0.78–1.33]; *p* = 0.92 with adjustment). CKD was more strongly associated with late (>1 year) postevent dementia (SHR 2.32 [1.70–3.17]; *p* < 0.001), particularly after TIA and minor stroke (SHR 3.08 [2.05–4.64]; *p* < 0.001), but not significantly so after adjustment (SHR 1.53 [0.90–2.60]; *p* = 0.12).

**Discussion:**

In patients with TIA and stroke, CKD was not independently associated with either pre- or postevent dementia, suggesting that renal-specific mechanisms are unlikely to play an important role in aetiology.

Chronic kidney disease (CKD) is associated with a significant burden of cognitive impairment that worsens with declining renal function.^1^ In the Reasons for Geographic and Racial Differences in Stroke (REGARDS) study, each 10 mL/min/1.73 m^2^ decrease in estimated glomerular filtration rate (eGFR) <60 mL/min/1.73 m^2^ was associated with an 11% increase in prevalence of cognitive dysfunction.^2^ Patients on hemodialysis are 3 times more likely to have severe cognitive impairment than age-matched patients not on dialysis with reported prevalence rates of 30%–40%.^3^

Both vascular and neurodegenerative hypotheses have been proposed to underlie CKD-related cognitive impairment.^4,5^ In support of the vascular hypothesis, there is a high prevalence of cardiovascular risk factors^6^ and a strong blood pressure–dependent association with stroke in CKD.^7^ Furthermore, impairment of executive function and processing speed are prominent in CKD, consistent with cerebrovascular disease.^8^ Although CKD may also augment neurodegeneration through the interplay of hypertension and Alzheimer pathology,^9^ or via high concentrations of uremic toxins such as neuroexcitatory guanidine compounds,^10^ any association between CKD and dementia might simply be due to increased cerebrovascular disease associated with CKD. For example, silent cerebral infarction is increased in patients with CKD^11^ and is associated with cognitive impairment.^12^ However, in one previous study, CKD was predictive of all-cause dementia independent of both previous symptomatic cerebrovascular disease and small vessel disease on brain imaging.^13^ We therefore aimed to determine the associations between CKD and all-cause dementia before and after TIA or stroke, with adjustment for measures of severity of the initial event, vascular risk factors, and other determinants of susceptibility in a large, longitudinal population-based study with standardized assessment of dementia to 5-year follow-up.

## Methods

Consecutive patients with TIA or stroke were prospectively recruited from April 1, 2002, to March 31, 2012, from the Oxford Vascular Study (OXVASC), an ongoing population-based study of all acute vascular events (including TIA, stroke, acute coronary syndromes, and peripheral vascular events). The study population comprises all 92,728 individuals, irrespective of age, registered with about 100 general practitioners (GPs) in 9 general practices in Oxfordshire, United Kingdom. The methodology of OXVASC was approved by the Oxfordshire Research Ethics Committee. Multiple methods of ascertainment are used to ascertain patients with TIA or stroke, as detailed elsewhere.^14^ Briefly, multiple overlapping methods of hot and cold pursuit are used to achieve near-complete ascertainment of all individuals with TIA or stroke and to minimize selection biases in determining dementia risk.^15^ These include a daily, rapid access TIA clinic to which participating GPs and the local emergency department (ED) refer all individuals with unhospitalized TIA or stroke, daily searches of ward admissions (medical, cardiology, stroke unit, neurology), ED attendance register and in-hospital bereavement office death records, and monthly searches of death certificates, coroner's reports (for out-of-hospital deaths), GP and hospital diagnostic/discharge codes, and brain/vascular imaging referrals. Written informed consent (or assent from relatives) was obtained for study interview and follow-up, including ongoing review of all primary care/hospital records and centralized health and death data. eAppendix 1 (links.lww.com/WNL/B712) provides further details on the study population and case ascertainment methodology.

Patients were assessed by a study physician as soon as possible after their TIA/stroke, usually within 1 or 2 days of the event. TIA and stroke were defined using WHO criteria^16^ (i.e., patients with infarction on brain imaging but symptoms lasting <24 hours were classed as TIA) with review of all cases by the same senior vascular neurologist (P.M.R.) throughout the study (see eAppendix 1, links.lww.com/WNL/B712, for definitions of events). A detailed clinical history was recorded in all patients by interview using a standardized questionnaire, including education; medical history and vascular risk factors were recorded in all patients, supplemented by primary care records (see eAppendix 1). Premorbid functional status was assessed using modified Rankin Scale and Barthel scores and stroke severity was assessed with the National Institutes of Health Stroke Scale (NIHSS) score. Baseline brain and vascular imaging and other investigations were performed as reported previously.^14,17^

CKD was defined as eGFR less than 60 mL/min/1.73 m^2^ for 3 or more months as per 2012 Kidney Disease: Improving Global Outcomes (KDIGO) guidelines.^18^ eGFR was estimated using the Chronic Kidney Disease Epidemiology Collaboration Equation (CKD-EPI). eGFR was then categorized into 5 groups based on modified CKD classification by the National Kidney Foundation–Kidney Disease Outcomes Quality Initiative: eGFR ≥90, 60–89, 30–59, 15–30, and <15 mL/min/1.73 m^2^. For the purpose of statistical analysis, eGFR ≥60 mL/min/1.73 m^2^ was used as the reference, as there were no poststroke dementia events for eGFR ≥90 mL/min/1.73 m^2^ in some of the subgroup analyses. Similarly, the eGFR 15–30 and <15 mL/min/1.73 m^2^ groups were combined as the individual numbers within each group were small.

For details on the brain imaging, see eAppendix 1, links.lww.com/WNL/B712*.* Assessments were made blind to clinical data. A qualitative scale was used (Oxford scale) based on the white matter disease severity score (absent, mild, moderate, or severe) of the Blennow scale for CT scans and a modified version of the Fazekas scale for MRI scans.^19^

Multiple methods of follow-up were used to reduce attritional biases in identification of dementia.^20^ Follow-up interviews were done by trained nurses or study physicians at 1 month, 6 months, 1 year, and 5 years. If clinic follow-up was not possible, patients were assessed at home or via telephone. Cognitive function was tested with the Mini-Mental State Examination (MMSE)^21^ and Montreal Cognitive Assessment^22^ at face-to-face interviews and with the telephone Montreal Cognitive Assessment and the Telephone Interview for Cognitive Status–modified.^23^

Dementia was defined as pre- or postevent according to whether the diagnosis was made before or after the index event, as described previously (see eAppendix 1, links.lww.com/WNL/B712).^15,20,24,25^ Briefly, pre-event dementia diagnosis was made using the following information: (1) baseline clinical assessment by study physician and discussion with relatives or other informant; (2) the presence of any dementia diagnosis, and related consultations and investigations, where available, in the primary care record, with hand-searching of the entire record including individual consultations, clinic letters, and hospitalization documentation. The diagnosis of pre-event dementia was made by an experienced consultant physician with subspecialty interest in dementia (S.T.P.) using DSM-IV criteria (eTable 1).

In patients without pre-event dementia, postevent dementia was diagnosed by S.T.P. using the same methodology (i.e., with clinical and cognitive assessment data and hand-searching of primary care records to death or 5-year follow-up).^15,20,24,25^ MMSE was done at each follow-up interview and dementia was diagnosed if MMSE was <24 and remained <24 for all subsequent follow-ups with fulfilment of other DSM-IV criteria including impairment of functional status.^15,20,24,25^ In patients who had problems that interfered with testing (e.g., dysphasia), incomplete testing (e.g., because of blindness), were followed up by telephone, could not be tested at the study interview (e.g., because of severe deafness) follow-up, or who did not have a follow-up assessment, dementia was diagnosed on the basis of study records where available and hand-searching of primary care, hospital, and death records based on DSM-IV criteria (eTable 1, links.lww.com/WNL/B712).^15,20,24,25^

### Standard Protocol Approvals, Registrations, and Patient Consents

Written informed consent was obtained from all patients (or guardians of patients) participating in the study (consent for research).

### Statistical Analysis

Descriptive statistics were used to summarize baseline characteristics stratified by CKD status and according to eGFR categories. Continuous data (SD) or median (interquartile range [IQR]) and categorical data (n [%]) were compared with Mann-Whitney *U* tests (or Kruskal-Wallis tests if multiple groups) and χ^2^ tests, respectively. Age-adjusted *p* values for difference were calculated using logistic and linear regression where appropriate.

Associations between CKD and pre-event dementia were determined by binary logistic regression to generate odds ratios (ORs) adjusted for (1) age, sex, and education (model 1) and (2) age, sex, education, and other factors previously reported as associated with pre-event dementia,^25^ including white matter disease, prior stroke, diabetes mellitus, and also index stroke event severity (NIHSS) and dysphasia occurring after the index stroke (model 2).

All analyses involving postevent dementia were done after exclusion of cases of pre-event dementia. Cumulative incidence of dementia postevent was calculated by Kaplan-Meier methods censoring at death as described previously.^25^ Cox regression was used to determine hazard ratios (HRs) for associations between overall 5-year risk of postevent dementia and separately for early (≤1 year) and late (>1 year) postevent dementia (patients with late dementia were excluded from analyses of early dementia and vice versa). Regression analyses were adjusted (1) for age, sex, and education (model 1); (2) for age, sex, education, and other factors reported as associated with dementia after TIA/stroke,^25^ including stroke severity (NIHSS), white matter disease, dysphasia, prior stroke, and diabetes mellitus; and (3) with further adjustment for baseline cognitive test score (model 3). Similar analyses were done restricted to TIA and minor stroke (defined as NIHSS <3 as per OXVASC protocol) and major stroke (NIHSS ≥3).^25^

We did not examine primary intracerebral hemorrhage (PICH) separately owing to small numbers with this stroke subtype, but we undertook sensitivity analyses for pre- and postevent dementia in which PICH was excluded. We performed further sensitivity analyses excluding recurrent stroke on follow-up in analyses of postevent dementia.

Given that death is a competing risk for dementia (i.e., it precludes its occurrence), we performed exploratory analyses using competing risks regressions using cumulative incidence function covariate analysis, similarly adjusted for the above covariates, to study the overall associations of CKD with postevent dementia, and, according to onset, early vs late postevent dementia.^33^ From these regressions, we generated subdistribution HRs (SHRs) for comparison as these are thought to be more informative of risk or prognosis whereas standard (cause-specific) HRs are thought to be better indicators of etiology.^34^

Results were considered significant at *p* < 0.05. Statistical analyses were performed using SPSS version 25.0 (SPSS Inc.) and Stata version 13 (Stat Corp.).

### Data Availability

Requests for access to data should be submitted for consideration to the OXVASC Study Director (peter.rothwell@ndcn.ox.ac.uk).

## Results

Of 2,305 patients recruited from 2002 to 2012, 1,133 (49%) were male, 688 (30%) had TIA, and 1,617 (70%) had stroke, of which 1,482 were ischemic stroke and 135 were PICH. Table 1 shows the baseline characteristics at the time of the event for all patients and according to CKD status. The median age was 76.9 years (IQR 66.9–83.9) and hypertension was the most prevalent risk factor, being found in 1,405 individuals (61%).

**Table 1:**
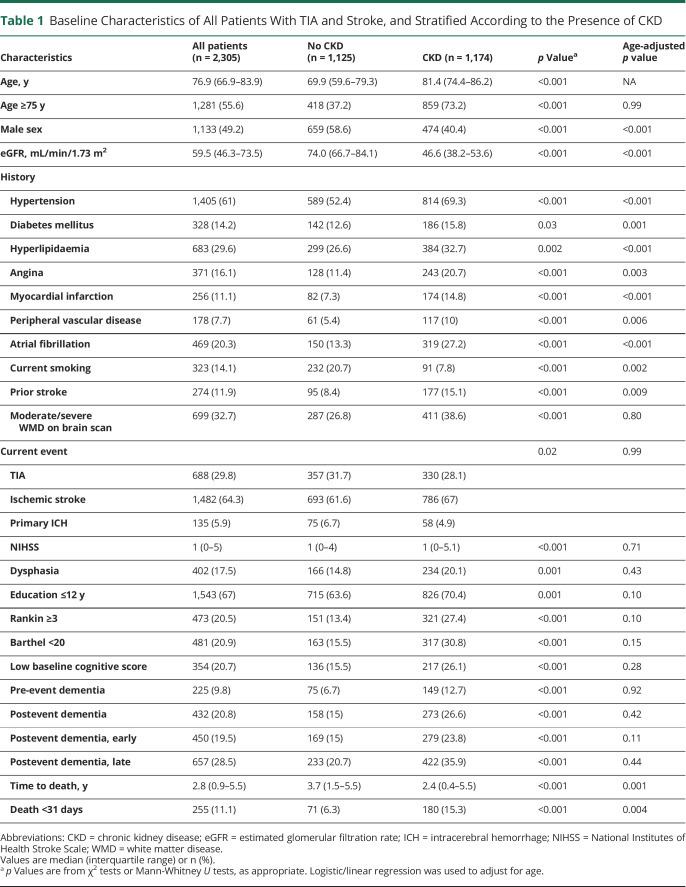
Baseline Characteristics of All Patients With TIA and Stroke, and Stratified According to the Presence of CKD

The median eGFR was 59.5 mL/min/1.73 m^2^. A total of 1,174 patients had CKD (50.9% of the study population, with eGFR of 30–59 mL/min/1.73 m^2^ in 1,040 [45.2%] and <30 mL/min/1.73 m^2^ in 134 [5.8%]). Only 12 patients (0.5%) were dialysis-dependent. Compared to those with normal renal function, the CKD group were older and had a higher burden of vascular risk factors and comorbidities including hypertension, diabetes mellitus, hyperlipidemia, ischemic heart disease, peripheral vascular disease, atrial fibrillation, and prior stroke. They were also more likely to have moderate to severe white matter disease, be less well-educated, and have a low baseline cognitive score. This burden of vascular and cognitive risk factors increased progressively with declining renal function, as evident when baseline demographics and comorbidities were examined according to eGFR categories (eTable 2, links.lww.com/WNL/B712).

Pre-event dementia was present in 225/2,305 (9.8%) patients. Compared with those with normal renal function, CKD was strongly associated with pre-event dementia on unadjusted analysis (OR 2.04, 95% confidence interval [CI] 1.52–2.72; *p* < 0.001) (Table 2), particularly at eGFR <30 mL/min/1.73 m^2^ (unadjusted OR 3.21, 1.96–5.26; *p* < 0.001). However, all associations attenuated and became nonsignificant after adjustment for age, sex, and education (model 1). After additional adjustment for other factors associated with pre-event dementia (model 2), there was further diminution of any association with CKD (OR 0.92, 0.65–1.31; *p* = 0.65; Table 2). Exclusion of PICH did not affect the results (Table 2). Stratifying by event severity showed broadly similar associations (Table 2).

**Table 2:**
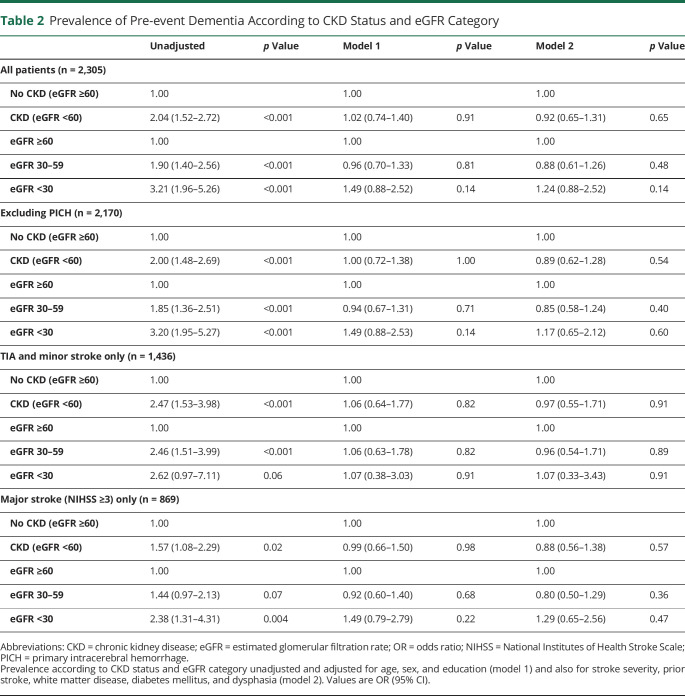
Prevalence of Pre-event Dementia According to CKD Status and eGFR Category

**Figure 1:**
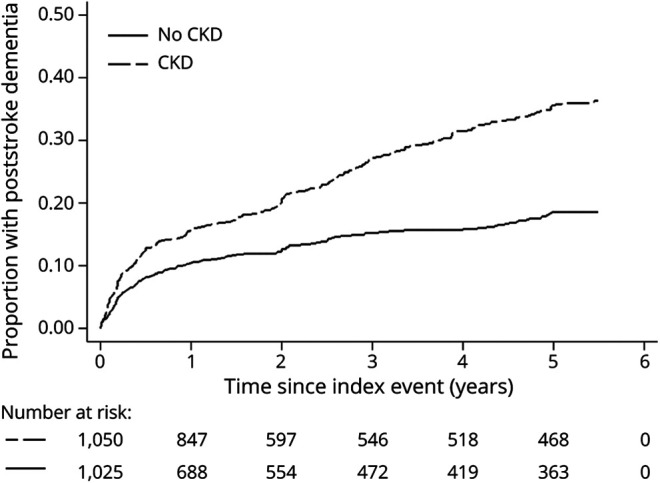
Kaplan-Meier (1 – Survival) Curve Showing the Cumulative Incidence of New Postevent Dementia (Excluding Pre-event Dementia) for All Patients (With and Without CKD) to 5-Year Follow-up CKD = chronic kidney disease.

Excluding those with pre-event dementia, during 7,721 patient-years of follow-up (median [IQR] 4.2 [1.5–5.5]), 432 patients developed postevent dementia (mean [SD] age 82.1 [8.7] years at diagnosis). There was a significantly greater risk of postevent dementia in patients with CKD compared to those with normal renal function (log-rank *p* < 0.001; Figure 1), and this was particularly marked in patients whose index event was a TIA or minor stroke (eFigure 1, links.lww.com/WNL/B712). However, although in unadjusted Cox regression analysis, CKD was strongly associated with postevent dementia to 5-year follow-up (HR 2.01, 95% CI, 1.65–2.44; *p* < 0.001 for all CKD), this association was also lost with adjustment for age, sex, and education, and other factors associated with postevent dementia (model 3 OR 1.09, 0.85–1.39; *p* = 0.50) (eTable 3). Results were similar when PICH was excluded and when the 194 patients with recurrent stroke on follow-up were excluded (eTable 3, links.lww.com/WNL/B712). CKD was more strongly associated with late (>1 year) vs early (<1 year) postevent dementia (HR 2.63, 1.96–3.53, *p* < 0.001 vs 1.60, 1.23–2.09, *p* = 0.001) (eTable 4, links.lww.com/WNL/B712), but the associations were also lost after adjustment (model 3, HR 1.40, 0.98–2.00, *p* = 0.06 for late dementia vs 0.90, 0.64–1.28, *p* = 0.57 for early dementia).

The results were qualitatively and quantitatively similar when analyses were repeated using competing risk regressions (Tables 3 and 4). CKD was again associated with postevent stroke in the unadjusted model (SHR 1.74, 1.43–2.12; *p* < 0.001) but not in the multivariate-adjusted models (SHR 0.97, 0.79–1.20; *p* = 0.77, 0.94, 0.76–1.17; *p* = 0.60; and 1.01, 0.78–1.33; *p* = 0.92, for models 1, 2 and 3, respectively) (Table 3). The crude associations between eGFR categories and postevent dementia were weaker than those from the cause-specific hazards model, particularly for eGFR <30 mL/min (SHR 1.51, 0.98–2.30 vs HR 2.48, 1.62–3.78). However, as per the cause-specific models, there were no independent associations between eGFR categories and postevent dementia after multivariate adjustment (Table 3). Restricting analysis to only ischemic or major stroke events produced similar results. Competing risk analysis for early (<1 year) vs late (>1 year) postevent dementia is shown in Table 4, where CKD is again more strongly associated with later events (SHR 2.32, 1.70–3.17; *p* < 0.001 vs 1.42, 1.10–1.83; *p* = 0.01) but the association did not reach significance after complete adjustment (SHR 1.47, 0.99–2.18; *p* = 0.06). CKD was particularly associated with late postevent dementia in the TIA and minor stroke subgroup (SHR 3.08, 2.05–4.64; *p* < 0.001), attenuating with adjustment for age, sex, education, stroke severity, prior stroke, white matter disease, diabetes, and dysphasia (SHR 1.70, 1.05–2.76; *p* = 0.03), and with additional adjustment for baseline cognitive score (SHR 1.53, 0.90–2.60; *p* = 0.12).

**Table 3:**
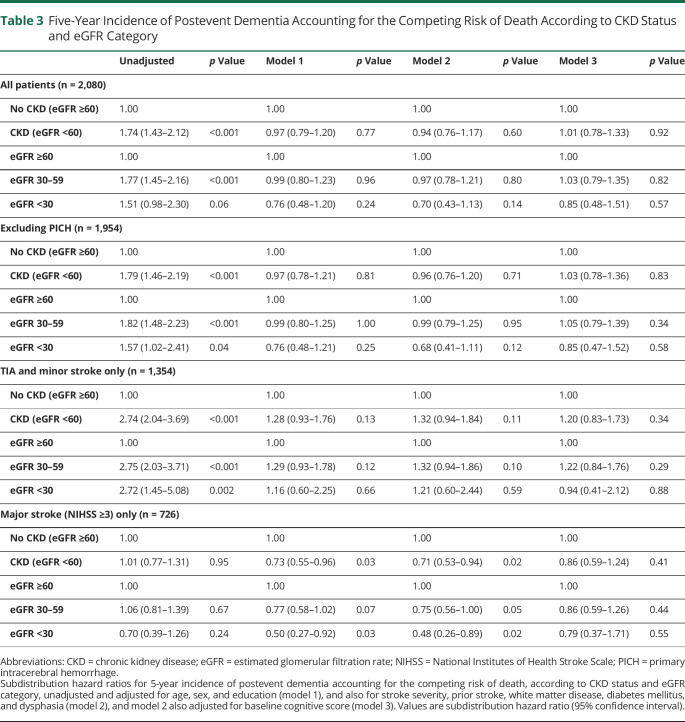
Five-Year Incidence of Postevent Dementia Accounting for the Competing Risk of Death According to CKD Status and eGFR Category

**Table 4:**
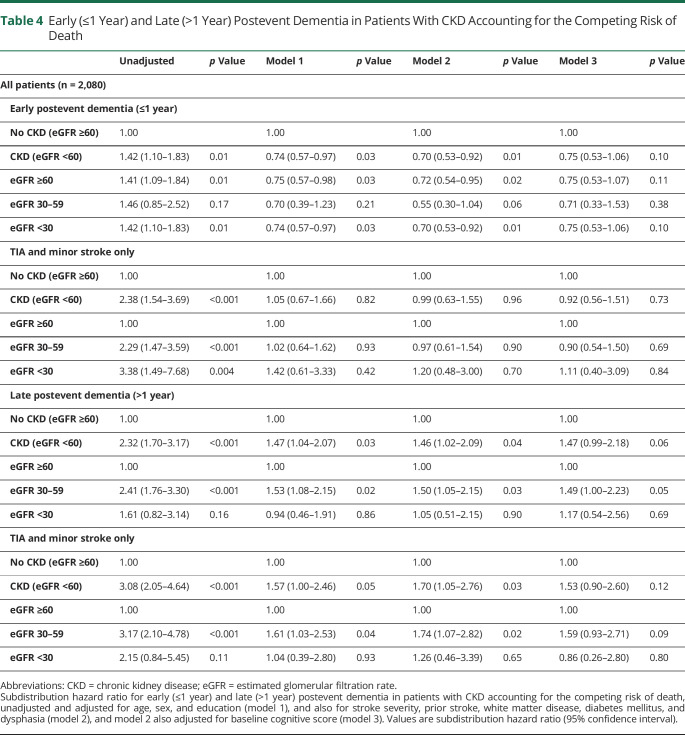
Early (≤1 Year) and Late (>1 Year) Postevent Dementia in Patients With CKD Accounting for the Competing Risk of Death

## Discussion

In a large, prospective, population-based cohort study, CKD was not independently associated with either pre- or postevent dementia in patients with TIA and stroke. Associations were greatly attenuated after adjustment for age, sex, education, and other factors previously shown to be associated with dementia including stroke severity and white matter disease,^25^ suggesting that renal-specific neurodegenerative mechanisms are unlikely to play an important role in the relationship between CKD and dementia, at least not in mild to moderate predialysis stages. However, the consistently higher prevalence rates of pre- and postevent dementia in patients with CKD does identify them as a vulnerable group.

Although CKD has been consistently associated with an increased risk of cognitive decline,^2,26,27^ we did not find a strong independent association with dementia in our study, even at greater levels of renal dysfunction or when restricted to major stroke events. There are a number of potential explanations for this discordance. First, most of the cognitive studies to date have focused on the relationship between CKD and mild cognitive impairment (MCI) rather than dementia.^28,29^ Second, in contrast to our study, where most patients had predialysis CKD and the majority had stage 3 (mild) CKD, previous dementia studies have been mainly dialysis-based.^30,31^ Dialysis patients have unique additional risk factors intrinsic to the dialysis procedure including cerebral hypoperfusion and this intradialytic hemodynamic instability causes transient cerebral stunning, leading to cumulative ischemic white matter changes and cognitive changes over time.^32^ Third, although we did not see an association between more advanced CKD and dementia, this is in keeping with the literature that suggests that duration of kidney disease or the rate of eGFR decline correlate better with cognitive dysfunction than severity of CKD.^33,34^

Mechanisms underlying the pathogenesis of MCI and dementia in CKD are poorly understood. It has been proposed that uremic neurotoxins interacting with neural progenitor cells, the brain vasculature, the glymphatic system, and monoaminergic neurons may play a role.^35^ High concentrations of uremic toxins such as tumor necrosis factor can impair synapse function and memory.^36^ The glymphatic clearance of waste products occurs primarily during sleep and CKD is associated with sleep disorders.^37^ In addition, high levels of the circulating phosphaturic hormone fibroblast growth factor 23 (FGF-23), often found in CKD, have been associated with incident dementia.^38^ However, the absence of an independent association between CKD and dementia in our patients with TIA/stroke questions any renal-specific mechanisms over and above the effects of CKD on cerebrovascular disease itself. For example, our associations were stronger for late (>1 year) postevent dementia, particularly in the TIA and minor stroke subgroup, in whom new vascular dementia is often related to recurrent strokes or progressive cerebral small vessel disease (SVD).^39^ Severe SVD has been implicated as the most important mechanism in late poststroke dementia^40^ and is strongly associated with CKD, especially at younger ages.^41^

In addition to the large study size, population-based design, and comprehensive adjustment for potential confounders, and detailed consideration of various biases in detection of dementia,^26-29^ a further strength of our study is the inclusion of a competing risk analysis to account for the risk of death—the importance of which is being increasingly recognized in survival analyses of CKD given the high baseline mortality rate.^42,43^ The results from the competing risk analysis were qualitatively and quantitatively similar to those from the Cox (cause-specific) proportional hazards model with the exception of the associations between advanced CKD (eGFR <30 mL/min/1.73 m^2^) and postevent dementia, which decreased. This likely reflects the stronger association between advanced CKD and the competing event (i.e., death) resulting in lower SHRs than expected,^44^ and is consistent with the findings in a previous study relating dialysis to risk for developing dementia.^43^

Our study has a number of limitations. First, TIA/stroke and dementia diagnoses were adjudicated by 2 investigators rather than by a consensus panel, but this ensured consistent diagnosis over the 15-year time period of this study. Second, pre-event dementia was retrospectively diagnosed, but the use of multiple sources should have minimized misclassification. Third, we did not study the relationship between CKD and specific subtypes of dementia because clinical classification is challenging in older patients and mixed pathology is the most common finding in neuropathologic studies.^45^ Moreover, most dementia occurring after stroke is coded as being of “unspecified” cause in routine practice.^35^ Fourth, we were unable to measure urine albumin excretion, which some studies suggest is more strongly and independently associated with cognitive dysfunction than eGFR.^46,47^ Fifth, as most of the CKD population had only mild to moderate disease, we may not have captured independent associations with dementia that may exist in later stages or in those who are dialysis-dependent. In addition, this was a highly select population enriched with vascular risk factors and therefore, the results may not be entirely generalizable to less multimorbid CKD. Sixth, as GFR was estimated from serum creatinine, this can lead to an overestimation of GFR in those with low muscle mass.^48^ Because muscle mass loss is also associated with cognitive decline,^49^ this may have underestimated the effect size of any association between CKD and cognitive decline. Our study should ideally be replicated using GFR estimated from cystatin-C molecule independent of muscle mass, which may increase precision of eGFR equations and has also been shown to colocalize with β-amyloid in the brain.^50^

Dementia is more common in patients with CKD than in the general population but predialysis CKD itself appears not to be independently associated with either pre- or poststroke dementia, except possibly with late-onset dementia in those with minor stroke events. Patients with CKD appear to have a clustering of risk factors associated with dementia including prestroke factors (advanced age, diabetes, and atrial fibrillation), stroke factors (greater event severity, dysphasia, and disability), and lower brain reserve (low education, premorbid dependency, and leukoaraiosis) that likely mediate much of the unadjusted relationship between CKD and dementia. Further studies are needed to determine if there are additional unique mechanisms or pathways leading to late-onset dementia in those with CKD and minor stroke events.

## Glossary

CI: confidence interval
CKD: chronic kidney disease
DSM-IV: *Diagnostic and Statistical Manual of Mental Disorders, 4th edition*
ED: emergency department
eGFR: estimated glomerular filtration rate
GP: general practitioner
HR: hazard ratio
IQR: interquartile range
MCI: mild cognitive impairment
MMSE: Mini-Mental State Examination
NIHSS: National Institutes of Health Stroke Scale
OR: odds ratio
OXVASC: Oxford Vascular Study
PICH: primary intracerebral hemorrhage
SHR: subdistribution hazard ratio
SVD: small vessel disease

## References

[1] Harhay MN, Xie D, Zhang X, et al. Cognitive impairment in non-dialysis-dependent CKD and the transition to dialysis: findings from the Chronic Renal Insufficiency Cohort (CRIC) study. Am J Kidney Dis. 2018;72(4):499-508. 29728316 10.1053/j.ajkd.2018.02.361 PMC6153064

[2] Kurella Tamura M, Wadley V, Yaffe K, et al. Kidney function and cognitive impairment in US adults: the Reasons for Geographic and Racial Differences in Stroke (REGARDS) study. Am J Kidney Dis. 2008;52(2):227-234. 18585836 10.1053/j.ajkd.2008.05.004 PMC2593146

[3] Murray AM, Tupper DE, Knopman DS, et al. Cognitive impairment in hemodialysis patients is common. Neurology. 2006;67(2):216-223. 16864811 10.1212/01.wnl.0000225182.15532.40

[4] Bugnicourt JM, Godefroy O, Chillon JM, Choukroun G, Massy ZA. Cognitive disorders and dementia in CKD: the neglected kidney-brain axis. J Am Soc Nephrol. 2013;24(3):353-363. 23291474 10.1681/ASN.2012050536

[5] Kelly D, Rothwell PM. Disentangling the multiple links between renal dysfunction and cerebrovascular disease. J Neurol Neurosurg Psychiatry. 2020;91(1):88-97. 31511306 10.1136/jnnp-2019-320526 PMC6952845

[6] Cheung AK, Sarnak MJ, Yan G, et al. Atherosclerotic cardiovascular disease risks in chronic hemodialysis patients. Kidney Int. 2000;58(1):353-362. 10886582 10.1046/j.1523-1755.2000.00173.x

[7] Kelly DM, Rothwell PM. Does chronic kidney disease predict stroke risk independent of blood pressure? A systematic review and meta-regression. Stroke. 2019;50(11):3085-3092. 31594463 10.1161/STROKEAHA.119.025442 PMC6824504

[8] Weiner DE, Scott TM, Giang LM, et al. Cardiovascular disease and cognitive function in maintenance hemodialysis patients. Am J Kidney Dis. 2011;58(5):773-781. 21778003 10.1053/j.ajkd.2011.03.034 PMC3199371

[9] Duron E, Hanon O. Vascular risk factors, cognitive decline, and dementia. Vasc Health Risk Manag. 2008;4(2):363-381. 18561512 10.2147/vhrm.s1839 PMC2496986

[10] De Deyn PP, Vanholder R, Eloot S, Glorieux G. Guanidino compounds as uremic (neuro)toxins. Semin Dial. 2009;22(4):340-345. 19708978 10.1111/j.1525-139X.2009.00577.x

[11] Kobayashi M, Hirawa N, Yatsu K, et al. Relationship between silent brain infarction and chronic kidney disease. Nephrol Dial Transpl. 2009;24(1):201-207. 10.1093/ndt/gfn419 PMC2639313 18697797

[12] Yao H, Araki Y, Takashima Y, Uchino A, Yuzuriha T, Hashimoto M. Chronic kidney disease and subclinical brain infarction increase the risk of vascular cognitive impairment: the Sefuri study. J Stroke Cerebrovasc Dis. 2017;26(2):420-424. 28341210 10.1016/j.jstrokecerebrovasdis.2016.10.002

[13] Miwa K, Tanaka M, Okazaki S, et al. Chronic kidney disease is associated with dementia independent of cerebral small-vessel disease. Neurology. 2014;82(12):1051-1057. 24553427 10.1212/WNL.0000000000000251

[14] Rothwell PM, Coull AJ, Giles MF, et al. Change in stroke incidence, mortality, case-fatality, severity, and risk factors in Oxfordshire, UK from 1981 to 2004 (Oxford Vascular Study). Lancet. 2004;363(9425):1925-1933. 15194251 10.1016/S0140-6736(04)16405-2

[15] Pendlebury ST, Chen PJ, Bull L, Silver L, Mehta Z, Rothwell PM. Methodological factors in determining rates of dementia in transient ischemic attack and stroke: (I) impact of baseline selection bias. Stroke. 2015;46(3):641-646. 25657179 10.1161/STROKEAHA.114.008043 PMC4342416

[16] A classification and outline of cerebrovascular diseases: II. Stroke. 1975;6(5):564-616. 1179466 10.1161/01.str.6.5.564

[17] Rothwell PM, Coull AJ, Silver LE, et al. Population-based study of event-rate, incidence, case fatality, and mortality for all acute vascular events in all arterial territories (Oxford Vascular Study). Lancet. 2005;366(9499):1773-1783. 16298214 10.1016/S0140-6736(05)67702-1

[18] National Kidney Foundation. K/DOQI clinical practice guidelines for chronic kidney disease: evaluation, classification, and stratification. Am J Kidney Dis. 2002;39(2 suppl 1):S1-S266. 11904577

[19] Simoni M, Li L, Paul NL, et al. Age- and sex-specific rates of leukoaraiosis in TIA and stroke patients: population-based study. Neurology. 2012;79(12):1215-1222. 22955138 10.1212/WNL.0b013e31826b951e PMC3440447

[20] Pendlebury ST, Chen PJ, Welch SJ, et al. Methodological factors in determining risk of dementia after transient ischemic attack and stroke: (II) effect of attrition on follow-up. Stroke. 2015;46(6):1494-1500. 25953366 10.1161/STROKEAHA.115.009065 PMC5117256

[21] Folstein MF, Folstein SE, McHugh PR. "Mini-mental state": a practical method for grading the cognitive state of patients for the clinician. J Psychiatr Res. 1975;12(3):189-198. 1202204 10.1016/0022-3956(75)90026-6

[22] Nasreddine ZS, Phillips NA, Bédirian V, et al. The Montreal Cognitive Assessment, MoCA: a brief screening tool for mild cognitive impairment. J Am Geriatr Soc*.* 2005;53(4):695-699. 15817019 10.1111/j.1532-5415.2005.53221.x

[23] Pendlebury ST, Welch SJ, Cuthbertson FC, Mariz J, Mehta Z, Rothwell PM. Telephone assessment of cognition after transient ischemic attack and stroke: modified telephone interview of cognitive status and telephone Montreal Cognitive Assessment versus face-to-face Montreal Cognitive Assessment and neuropsychological battery. Stroke. 2013;44(1):227-229. 23138443 10.1161/STROKEAHA.112.673384 PMC5593099

[24] Pendlebury ST, Klaus SP, Thomson RJ, Mehta Z, Wharton RM, Rothwell PM. Methodological factors in determining risk of dementia after transient ischemic attack and stroke: (III) applicability of cognitive tests. Stroke. 2015;46(11):3067-3073. 26463688 10.1161/STROKEAHA.115.010290 PMC5321486

[25] Pendlebury ST, Rothwell PM. Incidence and prevalence of dementia associated with transient ischaemic attack and stroke: analysis of the population-based Oxford Vascular Study. Lancet Neurol. 2019;18(3):248-258. 30784556 10.1016/S1474-4422(18)30442-3 PMC6390174

[26] Kurella M, Chertow GM, Fried LF, et al. Chronic kidney disease and cognitive impairment in the elderly: the Health, Aging, and Body Composition study. J Am Soc Nephrol. 2005;16(7):2127-2133. 15888561 10.1681/ASN.2005010005

[27] Madan P, Kalra OP, Agarwal S, Tandon OP. Cognitive impairment in chronic kidney disease. Nephrol Dial Transpl. 2007;22(2):440-444. 10.1093/ndt/gfl572 17023495

[28] Kurella Tamura M, Xie D, Yaffe K, et al. Vascular risk factors and cognitive impairment in chronic kidney disease: the Chronic Renal Insufficiency Cohort (CRIC) study. Clin J Am Soc Nephrol. 2011;6(2):248-256. 20930087 10.2215/CJN.02660310 PMC3052213

[29] Otobe Y, Hiraki K, Hotta C, et al. Mild cognitive impairment in older adults with pre-dialysis patients with chronic kidney disease: prevalence and association with physical function. Nephrology. 2019;24(1):50-55. 28949427 10.1111/nep.13173

[30] McAdams-DeMarco MA, Daubresse M, Bae S, Gross AL, Carlson MC, Segev DL. Dementia, Alzheimer’s disease, and mortality after hemodialysis initiation. Clin J Am Soc Nephrol. 2018;13(9):1339-1347. 30093374 10.2215/CJN.10150917 PMC6140560

[31] Kalirao P, Pederson S, Foley RN, et al. Cognitive impairment in peritoneal dialysis patients. Am J Kidney Dis. 2011;57(4):612-620. 21295896 10.1053/j.ajkd.2010.11.026 PMC3121243

[32] Findlay MD, Dawson J, Dickie DA, et al. Investigating the relationship between cerebral blood flow and cognitive function in hemodialysis patients. J Am Soc Nephrol. 2019;30(1):147-158. 30530658 10.1681/ASN.2018050462 PMC6317612

[33] Mendley SR, Matheson MB, Shinnar S, et al. Duration of chronic kidney disease reduces attention and executive function in pediatric patients. Kidney Int. 2015;87(4):800-806. 25252026 10.1038/ki.2014.323 PMC4372504

[34] Helmer C, Stengel B, Metzger M, et al. Chronic kidney disease, cognitive decline, and incident dementia: the 3C study. Neurology. 2011;77(23):2043-2051. 22116945 10.1212/WNL.0b013e31823b4765

[35] Viggiano D, Wagner CA, Martino G, et al. Mechanisms of cognitive dysfunction in CKD. Nat Rev Nephrol. 2020;16(8):452-469 32235904 10.1038/s41581-020-0266-9

[36] Yang G, Parkhurst CN, Hayes S, Gan WB. Peripheral elevation of TNF-α leads to early synaptic abnormalities in the mouse somatosensory cortex in experimental autoimmune encephalomyelitis. Proc Natl Acad Sci USA. 2013;110(25):10306-10311. 23733958 10.1073/pnas.1222895110 PMC3690863

[37] Kennedy C, Ryan SA, Kane T, Costello RW, Conlon PJ. The impact of change of renal replacement therapy modality on sleep quality in patients with end-stage renal disease: a systematic review and meta-analysis. J Nephrol. 2018;31(1):61-70. 28573387 10.1007/s40620-017-0409-7

[38] McGrath ER, Himali JJ, Levy D, et al. Circulating fibroblast growth factor 23 levels and incident dementia: the Framingham Heart Study. PLoS One. 2019;14(3):e0213321. 30830941 10.1371/journal.pone.0213321 PMC6398923

[39] Pendlebury ST, Rothwell PM. Prevalence, incidence, and factors associated with pre-stroke and post-stroke dementia: a systematic review and meta-analysis. Lancet Neurol. 2009;8(11):1006-1018. 19782001 10.1016/S1474-4422(09)70236-4

[40] Mok VC, Lam BY, Wong A, Ko H, Markus HS, Wong LK. Early-onset and delayed-onset poststroke dementia - revisiting the mechanisms. Nat Rev Neurol. 2017;13(3):148-159. 28211452 10.1038/nrneurol.2017.16

[41] Liu B, Lau KK, Li L, et al. Age-specific associations of renal impairment with magnetic resonance imaging markers of cerebral small vessel disease in transient ischemic attack and stroke. Stroke. 2018;49(4):899-904. 29523652 10.1161/STROKEAHA.117.019650 PMC5895118

[42] Findlay M, MacIsaac R, MacLeod MJ, et al. The association of atrial fibrillation and ischemic stroke in patients on hemodialysis: a competing risk analysis. Can J Kidney Health Dis. 2019;6:2054358119878719. 31632680 10.1177/2054358119878719 PMC6767723

[43] Kuo YT, Li CY, Sung JM, et al. Risk of dementia in patients with end-stage renal disease under maintenance dialysis: a nationwide population-based study with consideration of competing risk of mortality. Alzheimers Res Ther*.* 2019;11(1):31. 30967155 10.1186/s13195-019-0486-z PMC6456981

[44] Lau B, Cole SR, Gange SJ. Competing risk regression models for epidemiologic data. Am J Epidemiol. 2009;170(2):244-256. 19494242 10.1093/aje/kwp107 PMC2732996

[45] Schneider JA, Arvanitakis Z, Bang W, Bennett DA. Mixed brain pathologies account for most dementia cases in community-dwelling older persons. Neurology. 2007;69(24):2197-2204. 17568013 10.1212/01.wnl.0000271090.28148.24

[46] Joosten H, Izaks GJ, Slaets JP, et al. Association of cognitive function with albuminuria and eGFR in the general population. Clin J Am Soc Nephrol. 2011;6(6):1400-1409. 21566108 10.2215/CJN.05530610 PMC3109938

[47] Martens RJ, Kooman JP, Stehouwer CD, et al. Estimated GFR, albuminuria, and cognitive performance: the Maastricht study. Am J Kidney Dis. 2017;69(2):179-191. 27291486 10.1053/j.ajkd.2016.04.017

[48] Vinge E, Lindergård B, Nilsson-Ehle P, Grubb A. Relationships among serum cystatin C, serum creatinine, lean tissue mass and glomerular filtration rate in healthy adults. Scand J Clin Lab Invest. 1999;59(8):587-592. 10691049 10.1080/00365519950185076

[49] Chang KV, Hsu TH, Wu WT, Huang KC, Han DS. Association between sarcopenia and cognitive impairment: a systematic review and meta-analysis. J Am Med Dir Assoc. 2016;17(12):1164.e7-1164.e15. 10.1016/j.jamda.2016.09.013 27816484

[50] Levy E, Sastre M, Kumar A, et al. Codeposition of cystatin C with amyloid-beta protein in the brain of Alzheimer disease patients. J Neuropathol Exp Neurol 2001;60(1):94-104. 11202179 10.1093/jnen/60.1.94

